# Relative age effect in elite soccer: More early-born players, but no better valued, and no paragon clubs or countries

**DOI:** 10.1371/journal.pone.0192209

**Published:** 2018-02-08

**Authors:** John R. Doyle, Paul A. Bottomley

**Affiliations:** Business School, Cardiff University, Cardiff, Wales, United Kingdom; Universita degli Studi di Verona, ITALY

## Abstract

The paper analyses two datasets of elite soccer players (top 1000 professionals and UEFA Under-19 Youth League). In both, we find a Relative Age Effect (RAE) for frequency, but not for value. That is, while there are more players born at the start of the competition year, their transfer values are no higher, nor are they given more game time. We use Poisson regression to derive a transparent index of the discrimination present in RAE. Also, because Poisson is valid for small frequency counts, it supports analysis at the disaggregated levels of country and club. From this, we conclude there are no paragon clubs or countries immune to RAE; that is clubs and countries do not differ systematically in the RAE they experience; also, that Poisson regression is a powerful and flexible method of analysing RAE data.

## Introduction

Children born early in their school year enjoy a considerable advantage over those born later in the school year, simply by being up to 12 months older than later-borns in the same class. They do comparatively well in terms of academic attainment [1, 2], sport [3, 4], and in their emotional and social life [5, 6]. Such Relative Age Effects (RAEs) tend to persist longer than the natural ironing out of maturational advantages suggests they should. One possibility is psychological: they are due to positive feedback loops that ingrain a sense of positive self-esteem and can-do [7]. Another is organizational: there are watershed moments in life when doors are opened to those showing early signs of talent, more obviously evident in slightly older children.

European football club academies recruit boys often before they are 9 years old. This is a watershed moment because by being selected, the boys are effectively inducted into an elite environment. No effort is spared to accelerate their progress through intensive training and inter-academy competition. This hothousing ensures that the academy boys are soon so far ahead of their peers that turnover of personnel is minimal in subsequent years. Because football is such a public, social, scrutinised and discussed event, it is difficult to imagine a scenario in which talent could remain hidden for long. It follows that the RAE profile present at academy entry is likely to persist until the next watershed moment which occurs in the late teens, culminating in professional contracts being offered to the select few. Indeed, the same RAE patterns *are* found in between successive year groups in the academy, be this in Spain (Fig 6 in [8]); Belgium (Table 1 in [9]); or England (Table 1 in [3]).

These arguments suggest that watershed moments in childhood and youth engender notable RAE bias, which Cobley et al.’s [10] meta-analytic review confirms. In football, bias exists at both club and national levels for youths [11, 12] respectively; and in the professional game [13]. More worryingly, Helsen et al.’s [14] comparison of key European leagues, ten years apart, (2000 and 2010 seasons), found RAE bias had *increased* overall, most notably in Belgium, Denmark, England, Germany, Spain, and Sweden (see S1 Table).

Nevertheless, there is also evidence, first identified in Canadian ice hockey by Gibbs, Jarvis and Dufur [15], but confirmed in both Spanish [11] and Australian soccer [16], that the effects of RAE might fade as players get older, and even reverse (the "underdog hypothesis"). Similarly, the clear pattern of RAE as overrepresentation is not always mirrored when measures of earnings, skill and performance are examined. Findings about elite players have been equivocal. Bryson, Gomez and Zhang [17] showed that later-borns earn more in professional ice hockey, and Fumarco, Gibbs, Jarvis and Rossi [18] that they are also more productive. On the other hand, Fumarco and Rossi [19] found that later-borns earn less (in soccer). More research is needed to clarify why the oversampled early-borns do not always press home the advantage of a privileged start, on into adulthood.

One possibility follows from soccer talent being multi-dimensional. Larkin and O'Connor [20] identified seven attributes that a pool of highly qualified coaches considered "most important" in their evaluation of boys (*e*.*g*., first touch, 1 v 1, striking the ball, coachability), and a further ten that were considered "moderately important". Not only may these attributes, or any other set of attributes [21], be weighted differently in the overall evaluation of a player (for different positions, by different coaches, at different ages), but also each attribute may vary in the rate at which it advances over the course of a year's maturation. In a parallel research stream, this is what was found in the rates of development of classroom talents such as mathematics, reading, and so on [22], making each susceptible to different degrees of RAE bias. Furthermore, talents and attributes vary in their receptiveness to practice. This whole multiplicity of what really constitutes football talent may therefore provide the younger player an opportunity to follow compensating strategies in the kinds of sub-talents they must develop in order to be selected. This, in turn, means that the cohort-young may be qualitatively different, to some degree, from their older team-mates. Consequently, how the course of their careers works out may well differ between cohort-young versus cohort-old. However, the current state of RAE research is a long way from being able to flesh out these possibilities into a coherent and convincing picture.

Against this backdrop, Furley, Memmert and Weigelt [23] proposed an interesting comparison between the top 100 footballers (who should bear the trace of early selection) and the top 100 billionaires (who should not), predicting RAE bias among the former, but not the latter. Crossed with this comparison, they analysed RAE as measured by *frequency* versus RAE as measured by monetary *value*. The authors concluded that early-born players were both more populous and higher valued than later-born players; but there were no such differences for billionaires. However, other researchers have disputed these claims, and their grounds for reservation have broader implications for RAE analysis in general, and this paper in particular.

Fumarco and Gibbs [24] noted that the billionaires were drawn from various countries and US states, having many different definitions of the school year [25]. Thus to assume that all ages should be measured against the calendar year is misleading. However, to facilitate international competition, most countries do align their domestic football year with the calendar year, though there are exceptions (*e*.*g*., England, Japan, South Korea, parts of USA), which warrant careful consideration.

In re-analysing the same data, Loffing [26] concluded that the positively skewed nature of the transfer values had erroneously driven the results–the "Lionel Messi effect"–and violated assumptions of the t-test used to compare players born in the first half versus second half of the year. Loffing also raised some important issues regarding statistical best practice. For instance, that grouping players into just two (biannual) categories loses meaningful information and reduces statistical power to detect genuine effects [27, 28]. Likewise, if the expected RAE signature is a diminishing trend of representation over the cohort year, then why does RAE research so often ignore this information by using the Chi-squared test, which is inherently directionless, and therefore not only statistically blunt, but vulnerable to non-RAE signatures? Loffing argued that correlation and regression analyses would be more useful, and research that has done so, for instance by using Spearman’s rank correlation [19, 29], is a step in the right direction.

Developing this theme, we further improve statistical power by analysing two large international datasets of soccer players, and by using Poisson regression for count data. There are three main benefits. First, the Poisson estimating equations can meaningfully quantify the relative odds of a player being selected who is born on day 1 versus day 365 of the competition year, using the Index of Discrimination, I_D_, [22] (for a detailed justification, see S1 Table: Relative odds of selection). Second, by focusing on RAE as skew in frequency and RAE as skew in value (transfer value, games played), we can contribute to the underdog literature which has so far produced mixed results. Third, by analysing at a disaggregate level, not only do we heed Fumarco and Gibb’s [24] warning about choice of competition year, but we can test the generalisability of our findings across countries and clubs. In so doing, we also hope to identify paragon countries or clubs: "paragon" in being less susceptible to RAE bias, and from whom others may learn. Pep Guardiola, then manager of Barcelona and himself a product of their academy, appeared optimistic when speaking about Barcelona's ability to identify talent, “having the technical eye to bypass things like age and size” [30], with the implication that they might be such a paragon club. And if not Barcelona, might it be Ajax, or Sporting Lisbon, as two other highly respected academies? Sadly, our data paint a different picture.

## Study 1: Top 1000 professional footballers

We collected information on the 1000 most valuable footballers currently playing professional soccer around the world from the database TransferMarkt [31] in which informed users “crowd-source” estimates of players’ transfer value. Herm, Callen-Bracker and Kreis [32] have shown that TransferMarkt provides an accurate reflection of real market values, including one-club players who have never been transferred. Aggregated over the 1000 players, transfer value totalled an estimated €11.6bn worth of talent.

What defines the competition year is a little fuzzy in this or any equivalent dataset. For most players, the competition year they grew up in was 1^st^ Jan. to 31^st^ Dec., but in many European countries it was 1^st^ Aug. to 31^st^ Jul. up until the late 1990s. Many older players in our dataset were first recruited into football academies under that premise, so a trace of that bias may still exist in this data. Moreover, some countries continue to maintain their own idiosyncratic competition years (*e*.*g*., England, Japan), while other smaller nations have unknown structures. We therefore first analyse the data using 1^st^ Jan.–31^st^ Dec. as the competition year for all players, acknowledging that any exceptions will create extraneous noise to dilute the signal of RAE bias. But for a cleaner signal, we also analyse players from the major footballing nations that have calendar year structure. These comprise about 2/3 of the 1000 players.

## Results

### RAE by frequency: Simple tests of mean values

For the sake of compatibility, all analyses and graphical displays are done on a weekly basis. Let a player’s birth-week number (W_B_) denote the *week* in which he was born: week 1 = 1^st^–7^th^ Jan.; week 2 = 8^th^–14^th^ Jan., and so on. Players born on days 365, and 366 in a leap year were included in week 52, with the frequency deflated by 7 / 8.25. Alternative (more complicated) methods of resolving the partial week 53 yielded almost identical results. W_B_ can then be transformed into time of birth, measuring how far through the competition year a player’s birthday is: *t*_*B*_ = (W_B_− 0.5) / 52. That is, each player's birth was notionally located at the midpoint of the week in which he was born (W_B_− 0.5). These numbers were then scaled to lie in the one year interval (0, 1).

Table 1 shows that the top 1000 players' mean birth-time ${\bar{t}}_{B}$ = 0.445 (corresponding to being born within week 23 of the year) was significantly lower than the null hypothesis of .50 associated with a uniform distribution across the year (*p* < .001); Wilcoxon’s one-sample test confirmed this (*p* < .001). Thus, the top 1000 footballers currently playing were born earlier in the competition year than expected by chance. While there is a small amount of seasonality in birth patterns in the general population, they tend to favour late summer births, suggesting that our results are conservative.

**Table 1:** RAE by frequency: Overall, and country-by-country analysis.

	t-test of mean				Poisson regression		
Country	N	${\bar{t}}_{B}$	p	b_0_	b_1_	R^2^	I_D_
Argentina	62	0.351	< .001	0.9592	-1.8513	0.21	6.37
Netherlands	41	0.386	< .05	0.3701	-1.3781	0.09	3.97
Turkey	23	0.390	< .05	-0.2168	-1.3557	0.07	3.88
Belgium	31	0.405	< .05	-0.0143	-1.1154	0.06	3.05
Spain	102	0.408	< .01	1.1654	-1.0880	0.13	2.97
Italy	72	0.420	< .05	0.7775	-1.0200	0.09	2.77
Germany	67	0.428	< .05	0.6341	-0.8246	0.07	2.28
Portugal	31	0.431		-0.1486	-0.7969	0.03	2.22
Brazil	100	0.440	< .05	0.9750	-0.6884	0.07	1.99
Russia	24	0.445		-0.4788	-0.6285	0.02	1.87
France	81	0.461		0.6718	-0.5057	0.03	1.66
All countries	1000	0.445	< .001	3.2566	-0.6466	0.37	1.91
Above 11 countries	634	0.418	< .001	2.9455	-0.9810	0.45	2.67

N is the number of players appearing in the top 1000 by transfer value; ${\bar{t}}_{B}$ is the mean time of birth (0 at beginning of year, 1 at end, 0.5 mid-year); Poisson estimating equation is y = exp(b_0_ + b_1_.t_B_); R^2^ is McFadden’s pseudo-R^2^; I_D_ is the Index of Discrimination = exp(-b_1_); p is the statistical significance for rejecting the null hypothesis ${\bar{t}}_{B}$ ≥ 0.50. Same significance levels also found when testing null hypothesis b_1_ ≥ 0.

Next, we repeated the analysis country-by-country for the 11 nations that contributed most players to the dataset *and* operated the 1^st^ Jan. to 31^st^ Aug. competition year. Again, each country’s birth-time distribution was found to be asymmetric and positively skewed, mean time of birth ${\bar{t}}_{B}$ < .50, with directional support in Portugal, France, and Russia, and statistically significant differences in eight other countries (all *p*’s < .05 for 1-tailed tests). Aggregating across these same nations, ${\bar{t}}_{B}$ = 0.418, *p* < .001 by both t-test and Wilcoxon's.

In summary, and consistent with prior research examining RAE among professional footballers, when assessed by *frequency*, birth-time distributions are notably asymmetric, favouring more early-born than later-born players [14]. This message is somewhat blunted, though still present, if *all* nations are analysed, erroneously assuming they all used the calendar competition year (${\bar{t}}_{B}$ = 0.445 versus ${\bar{t}}_{B}$ = 0.418). But, while disaggregated analyses can be illuminating, the mean birth-time statistic itself lacks transparency. For example, if ${\bar{t}}_{B}$ = 0.351 instead of 0.461, how much more disadvantaged are later-born players in Argentina compared with France? We now reanalyse the data with Poisson regression, which provides an answer to this question.

### RAE by frequency: Poisson regression on weekly counts

Poisson regression is a standard method for analysing low count data [33]. It explains the frequency count of an event (y) by an explanatory variable x, using the relationship: y = e^(b0 + b1x)^. Here y is frequency of birth in a given week, and x is where that week is in the competition year, as measured by t_B_. Over the course of a year RAE anticipates a declining number of players born each week. Having already rejected the hypothesis that mean time of birth ${\bar{t}}_{B}$ ≥ .50 using simpler statistical tests, this analysis would be strictly unnecessary if our only goal were to confirm the existence of RAE. However, Poisson regression yields an estimating equation from which we can derive the Index of Discrimination, I_D_ [22]. In contrast to ${\bar{t}}_{B}$, I_D_ is highly interpretable, managerially useful, and easy to calculate from the Poisson regression equation.

**Fig 1:**
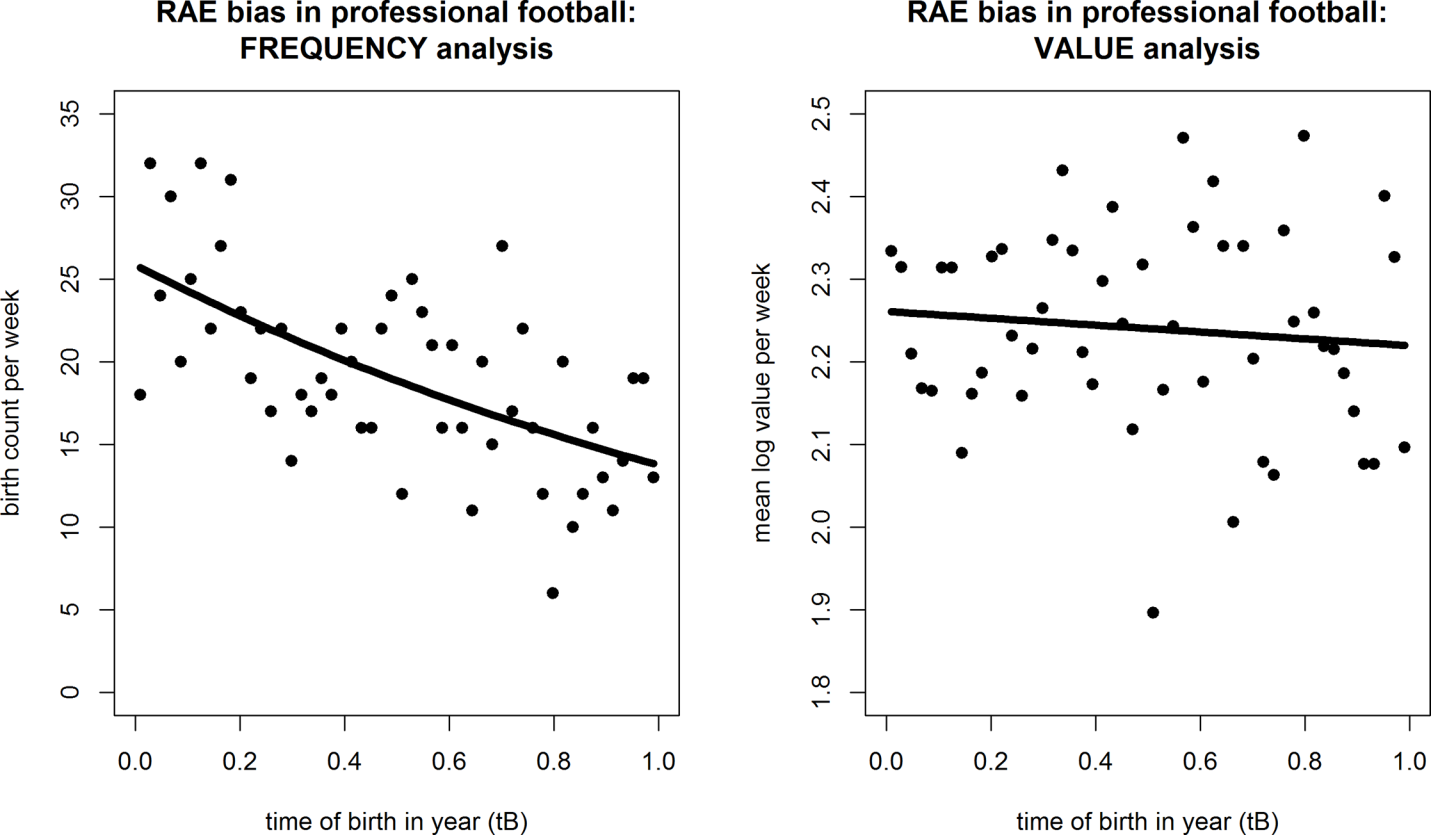
Scatterplots of RAE by frequency and value: 1000 top professional footballers. Left panel: Birth frequency by week of year (Poisson regression, best fit). Right panel: Mean log transfer value (€m.) by week (OLS regression, best fit).

A scatterplot relating birth frequency to *t*_*B*_ is given in the left-hand panel of Fig 1. The Poisson regression equations, fit statistics, and I_D_s are presented in the last four columns of Table 1. For the overall model using N = 1000, for instance:

$$Frequency=e^{\left(3.2566-0.6466t_{B}\right)}=e^{3.2566}\;e^{-0.6466t_{B}}=25.9611e^{-0.6466t_{B}}$$

The slope coefficient for *t*_*B*_ (time of birth) was significantly different from zero (*p* < .001), and the model explained 37% of the variation (McFadden’s pseudo R^2^). Adding the quadratic term t_B_^2^ did not add significantly to the model. This was found in both the Poisson count (here) and OLS value analyses; and in both Studies 1 and 2. They have therefore not been reported explicitly. Also, the dispersion coefficient was 1.05, indicating that the "variance ≈ mean" condition for Poisson is met, with no need for more complicated, variance-adjusting models such as quasi-poisson or negative binomial. Substituting into the equation values of *t*_*B*_ = 0 and *t*_*B*_ = 1 for the selection cut-off dates, gives expected player frequencies of 25.96 and 13.60, respectively. Taking a ratio of these extremes, we calculated the Index of Discrimination (I_D_) as 25.96 / 13.60 = 1.91. More simply, I_D_ is just e^-b1^, here e^+0.6466^. Similarly, I_D_ for the top 11 calendar year nations is 2.67. Players born right at the start of their nation's competition year are therefore nearly three times more likely to reach the top echelons of football than players born right at the end. So the I_D_ statistic highlights the age discrimination inherent in RAE in a manner that is not immediately obvious from mean birth-time ${\bar{t}}_{B}$ alone. Being born just after midnight on New Year's Eve, rather than just before can make a dramatic difference to a player’s sporting opportunities.

To assess the generalisability of this finding, we repeated the Poisson analysis on each of the 11 top nations individually. Reassuringly, there was good agreement between both sets of analyses (Poisson and t-tests), in Table 1, showing our results are not method-specific. Nonetheless, one advantage of Poisson regression is that the derived relative odds ratio I_D_ makes the disadvantage inherent in RAE so much more transparent and easily interpretable than examining ${\bar{t}}_{B}$ alone.

At first glance, in Table 1 there seem to be notable differences between countries. For instance, players born right at the start of the competition year were 6.37 times more likely to appear among the top 1000 players than those born right at the end, if they were Argentinean; but only 1.66 times more likely, if they were French. However, these differences are deceptive, indeed illusory. When an interaction term of (t_B_ x country) was added to the Poisson equation, it did not add significantly to the simpler model (*F* < 1). In other words, countries' slope coefficients did not differ significantly from each other, meaning that the effect of birth week was effectively the same across all eleven countries. Therefore, the seemingly large differences in I_D_ are what we would expect from the sampling distribution of the simple model (*i*.*e*., they are random differences in birthdays), rather than systematic differences in RAE between countries. This in turn suggests that there are no paragon countries, whose practices could be examined to discover why they are less susceptible to RAE. All countries are equally as bad as each other in this respect.

### RAE by transfer value

By contrast, there was no tendency for players born earlier in the competition year to be higher valued, which is readily apparent when comparing left and right-hand panels of Fig 1. The y-axis of the left panel shows, for each of the 52 weeks of the year, the *number* of players with birthdays falling in that week. The x-axis is the point in the year when each week occurs: *i*.*e*., measured by t_B_, lying in (0, 1). Meanwhile, the y-axis of the right panel shows the *mean log transfer value* (€m.) of those players born in each of the 52 weeks.

It is clear from the scatter of points themselves, and the superimposed regression relationship of best fit (in the left panel, Poisson regression to explain frequency counts per week; in the right panel, OLS regression to explain mean value per week), that there is: (i) a downward trend for player frequency with birth-week over the year; but (ii) no similar trend between players’ monetary value and birth time. To verify this finding, the analysis was replicated using various transformations of the player value data before forming the weekly averages, including log (as shown), reciprocal, ranked, and raw. But, the result remained unchanged (all model *F*s < 1).

In conclusion, analysis of the birth dates of the top 1000 professional football players reveals there is a downward trend in birth-frequency over the year. Just as clearly, there is no comparable trend in player value. While many more early-born rather than later-born players appear in the dataset, these players are no more highly valued in monetary terms, and by inference, no more skilful or talented.

## Study 2: UEFA Youth League

To replicate the findings of a RAE bias *by-frequency*, but none *by-value*, we turn to an equally large player dataset concerning the UEFA 2014–15 Youth League. All 32 clubs that competed in the UEFA Champion's League entered a parallel competition for their under 19 teams. Specifically, the sample comprised 1038 players whose country of origin matched that of their club. Non-domestic players (170) were excluded because we could not be certain about the competition year they had grown-up in [21]. The four English clubs were analysed using 1^st^ Sep. to 31^st^ Aug. as the competition year. All others were analysed using 1^st^ Jan. to 31^st^ Dec.

There are three reasons for this study. First, we replicate the principal findings from Study 1 on a second dataset. It is especially important that researchers ensure a thorough attempt has been made to reject any null hypothesis if they then wish to claim that they cannot, and thus claim here that no value effect is present. Second, we can assess whether RAE bias exists among all academies and to a similar extent, or whether some clubs have developed procedures that mitigate its effects, thereby addressing Pep Guardiola’s claim about Barcelona’s academy. Third, the dataset offers a glimpse of how RAE is likely to manifest itself in the next generation of European footballers playing at the highest levels.

## Results

### RAE by frequency

Again, the overall mean time of birth ${\bar{t}}_{B}$ = 0.345 was significantly lower than the null hypothesis of ${\bar{t}}_{B}$ = 0.50 (*p* < .001 using a one sample t-test), suggesting that player birth-dates did not fall uniformly, but were highly positively skewed, favouring early-born players. A Wilcoxon one-sample test confirmed this (*p* < .001). All 32 academies exhibited RAE bias (${\bar{t}}_{B}$ < 0.50), and these differences were statistically significant in 30 (out of 32) cases, with Arsenal and Maribor the exceptions.

To better understand the advantage to players born early in the competition year, we ran the corresponding Poisson regressions. Results are presented in Table 2. For the pooled model, the player frequency-by-week equation was:

$$Frequency=e^{\left(3.8234-1.9738t_{B}\right)}=e^{3.8234}\;e^{-1.9738t_{B}}=45.7595e^{-1.9738t_{B}}$$

with a pseudo R^2^ = 0.89, *p* < .001. Again, the dispersion coefficient was 0.96, close to 1, indicating that the "variance ≈ mean" condition for Poisson was met, with no need for more complicated, variance-adjusting models. The Index of Discrimination I_D_ = 7.20 (= 45.76 / 6.36) was notably higher than found with the top 1000 professional footballers (1.95, or 2.67). However, this is consistent with growing evidence which suggests that the advantages of early-birth may fade as: (i) players get older, or (ii) players advance to higher echelons in professional sport [11, 15]. The decrease of I_D_ from 7.20 at youth level to 2.67 at senior level also implies is that, *in the specific transition from youth (U19) to senior levels*, those cohort-younger players *who have got as far as U19* are actually advantaged over their older contemporaries. The italicised qualifying clauses should be particularly noted, because over the entire selection process from young boy to man, the cohort-young are still disadvantaged.

**Table 2:** RAE by frequency: Overall, and club-by-club analysis.

		t-test of mean			Poisson Regression			
Club	N	${\bar{t}}_{B}$	p	b_0_	b_1_	p	R^2^	I_D_
Galatasaray AS	27	0.275	< .001	0.5269	-3.1170	< .001	0.287	22.58
Liverpool*	30	0.283	< .001	0.5882	-2.9590	< .001	0.266	19.28
Barcelona	32	0.284	< .001	0.6511	-2.9534	< .001	0.293	19.17
Sporting Lisbon	31	0.294	< .001	0.5659	-2.7681	< .001	0.269	15.93
Chelsea*	30	0.296	< .001	0.5273	-2.7486	< .001	0.278	15.62
Juventus	27	0.305	< .001	0.3737	-2.5880	< .01	0.227	13.30
Malmo FF	40	0.305	< .001	0.7663	-2.5865	< .001	0.252	13.28
Shakhtar Donestsk	37	0.308	< .001	0.6751	-2.5434	< .001	0.253	12.72
Bayer Leverkusen	35	0.313	< .001	0.5949	-2.4640	< .001	0.237	11.75
Zenit St Petersburg	27	0.315	< .001	0.3242	-2.4283	< .01	0.207	11.34
Basel 1893	25	0.323	< .001	0.2099	-2.3112	< .01	0.169	10.09
Borussia Dortmund	31	0.328	< .01	0.3994	-2.2324	< .01	0.190	9.32
Real Madrid	35	0.329	< .001	0.5158	-2.2174	< .001	0.256	9.18
Athletico Madrid	31	0.332	< .001	0.3809	-2.1764	< .01	0.167	8.81
CSKA Moskva	31	0.335	< .001	0.3624	-2.1209	< .01	0.177	8.34
Olympiacos	39	0.340	< .001	0.5686	-2.0518	< .001	0.193	7.78
Bate Borisov	27	0.340	< .01	0.2004	-2.0502	< .01	0.153	7.77
Ajax	37	0.341	< .001	0.5099	-2.0339	< .01	0.208	7.64
Porto	35	0.344	< .01	0.4393	-1.9900	< .01	0.169	7.32
RSC Anderlecht	36	0.348	< .001	0.4499	-1.9392	< .01	0.193	6.95
Schalke 04	35	0.351	< .01	0.4064	-1.8954	< .01	0.162	6.66
Manchester City*	19	0.365	< .05	-0.2749	-1.6985	< .05	0.089	5.47
Paris Saint Germain	36	0.367	< .01	0.3535	-1.6695	< .01	0.141	5.31
AS Roma	31	0.369	< .01	0.1949	-1.6447	< .01	0.106	5.18
AS Monaco	29	0.371	< .05	0.1170	-1.6144	< .05	0.117	5.02
Atheletic Bilboa	40	0.372	< .01	0.4330	-1.5995	< .01	0.142	4.95
SL Benfica	29	0.380	< .05	0.0699	-1.4892	< .05	0.098	4.43
Bayern Muenchen	26	0.384	< .05	-0.0575	-1.4416	< .05	0.086	4.23
Ludogorets Razgrad	40	0.419	< .05	0.1927	-0.9912	< .05	0.045	2.69
Apoel	39	0.421	< .05	0.1536	-0.9585	< .05	0.052	2.61
NK Maribor	38	0.423		0.1212	-0.9432		0.047	2.57
Arsenal*	33	0.436		-0.0927	-0.7736		0.027	2.17
All 32 clubs	1038	0.345	< .001	3.8234	-1.9738	< .001	0.894	7.20

N is the number of “domestic” players appearing in the squad; ${\bar{t}}_{B}$ is the mean time of birth (0 at beginning of year, 1 at end, 0.5 mid-year); Poisson estimating equation is y = exp(b_0_ + b_1_.t_B_); R^2^ is McFadden’s pseudo-R^2^; I_D_ is the Index of Discrimination = exp(-b_1_); First p is the statistical significance for rejecting the null hypothesis ${\bar{t}}_{B}$ ≥ 0.50. Second p is the statistical significance for rejecting the null hypothesis b_1_ ≥ 0.

Asterisk (*) is for clubs using 1^st^ Sep. to 31^st^ Aug. competition year

All clubs did exhibit RAE bias to some degree. But, there were again striking differences as to the favourability associated with being early-born in the competition year. Players born right at the very start of the year were nearly 20 times more likely than those born at the very end to be recruited by academies at Galatasaray (22.58), Liverpool (19.28) and Barcelona (19.17), but only twice as likely if selected by Arsenal (2.17), Maribor (2.57), Apoel (2.61), or Ludogorets (2.69). Interestingly, Barcelona has one of the highest I_D_s, while at the same time it is celebrated as being a paragon academy, having nurtured the talent of Xavi, Iniesta, and Messi, in defiance of their small stature (biological maturity). Contrary to Pep Guardiola’s assertion in our introduction, the empirical evidence would suggest that Barcelona is therefore no paragon club when it comes to RAE bias in decisions of academy player selection.

However, these seemingly impressive inter-club differences were illusory rather than systematic, because when a club x t_B_ interaction was added to the Poisson equation, it was non-significant (*F* < 1), exactly as in the countries analysis. Thus, clubs did not differ significantly from each other in their RAE slopes, meaning that clubs' RAEs are best viewed as random samples drawn from a single distribution (that of the simple Poisson model). In conclusion, there are no paragon clubs; neither Barcelona, Ajax, Sporting Lisbon or any other. They are all essentially as biased as each other.

Since this is such a surprising result, further research is clearly needed before we can unequivocally conclude that there are no inter-club differences. For instance, if the degree of per-club bias (Table 2) persists year-on-year, idiosyncratically for each club, the bias would be systematic: if there is no relationship between each club’s level of bias year-on-year, the bias would be random in the sense argued here from non-significant interaction terms.

### RAE by value

Being at the start of their career, most players do not yet have monetary values, so we use games played as a proxy measure. Games played can signal a player's value in two different ways: by being chosen above team-mates, and / or by being talented enough to play for better teams that progress further in the competition.

**Fig 2:**
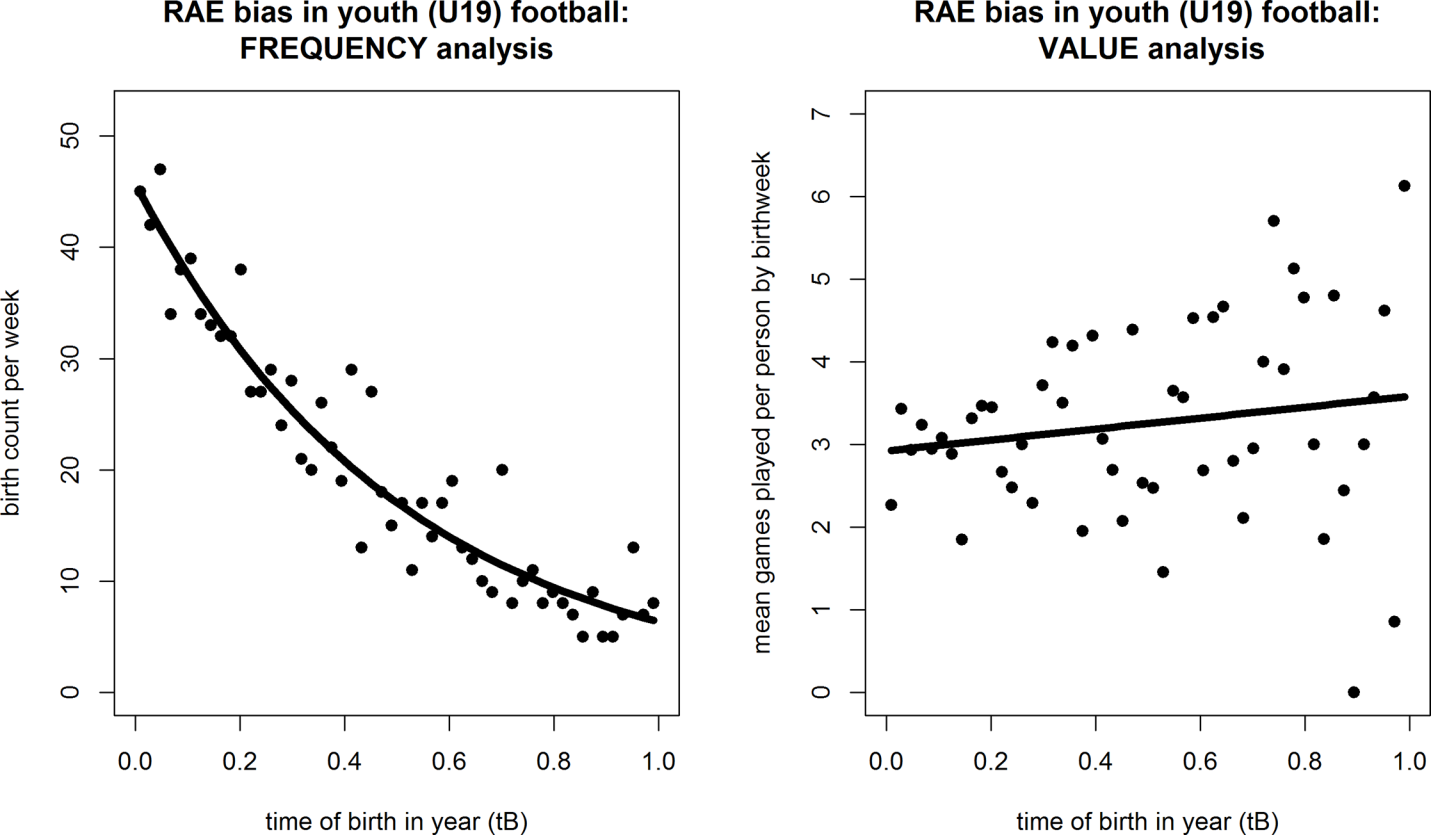
Scatterplots of RAE by frequency and value: 32 clubs’ U19 academy footballers. Left panel: Birth frequency by week of year (Poisson regression best fit). Right panel: Mean games played per player by birthweek (OLS regression best fit).

Fig 2 present the scatterplots and lines of best fit using Poisson (by frequency) and OLS (by value) regressions. As in Study 1, the value-analysis regression was not significant (*F* < 1), whether player weekly averages were expressed in terms of logs (as shown), reciprocal or ranked transformations. The flat slope of the line makes this abundantly clear (right panel).

In summary, these results match those we found with professional footballers. Figs 1 and 2 both tell much the same story: a downward trend over the cohort year for player frequency that is not matched by any trend for value. In essence, both clubs and countries selection policies appear to favour those born early in the competition year, but early-born players are no more skilled or talented than their later-born peers.

## Discussion and conclusions

Having analysed two large football player datasets we conclude, consistent with Cobley et al.’s [10] meta-analytic review, that there are advantages to being early-born, close to the competition year’s cut-off date, in that leading European football clubs and successful international nations both recruit more early-born players. However, our analyses also show that early-born players turn out to be no more talented or skilful, when measured by transfer value or games played.

We found no real evidence for paragon countries or paragon clubs in terms of their RAE (Relative Age Effect) profile. Indeed, Barcelona, often cited as having a paragon development centre, had one of the largest Index of Discrimination (I_D_) of all. But, it is possible these elevated levels are a symptom of their success. The TTG model of RAE [22] predicts that the more elitist the selection, the greater the RAE bias. Perhaps clubs with the most RAE bias, such as Barcelona, are drawing on a much larger population of eligible boys, leading to more elitist selection. However, empirical studies are few, though Schorer et al.’s [4] comparison of 49 European footballing nations found that differences in RAE were not related to depth of competition, measured by population size or ratio of domestic to total players. If RAE is inevitable, and clubs powerless to avoid it when making player selection decisions [34], then perhaps football authorities will have to implement corrective policy.

One methodological innovation in this paper is to use Poisson regression which, despite being the standard statistical technique to analyse low frequency count data, remains underused in RAE research. From it, we derive an easily interpretable measure of RAE bias, I_D_. Standard practice of using Chi-squared tests to show that observed quarterly or monthly frequencies differ from a uniform distribution is limited, and can even be misleading. RAE anticipates a decline in frequency across the year. Yet Chi-squared tests could be significant if the relationship were a U, an inverted-U, or an increasing frequency across the year ‒ none of which has an RAE signature. Also, with Poisson regression, we can legitimately perform more disaggregated analyses, and thereby build a more nuanced understanding of the RAE phenomena. Finally, Poisson regression can model more than one explanatory variable in the same analysis: for instance, to disentangle the effects of birthtime in the competition year (t_B_) from background variations in the population birthrate across the year; to model time-trends in RAE; or model mixed cohort definitions, as when school year and sports year are different; or to model interaction terms. As an example, in this paper we were able to show that clubs and countries are drawn from the same population pool when it comes to RAE, as we found no (country x t_B_), nor (club x t_B_) interactions, meaning that there is no evidence that paragon clubs or countries exist which are *reliably* less susceptible to RAE.

However, RAE as measured by player value tells a different story. Contrary to recent studies by Ashworth and Heyndels [29], Fumarco, Gibbs, Jarvis and Rossi [18], and McCarthy, Collins and Court [35], we found no evidence for a reversal of RAE; no upward trend in player value with later birth. Later-born players did not command higher transfer values, nor in youth football did they enjoy more playing opportunities. Nor indeed was there any evidence for an inverted U relationship in player value over the year, as in “the educated underdog hypothesis” [36], such that those in the middle of the year gain longer-term benefit from having to work harder to keep up with the older boys, whereas for the youngest, the competition is just too strong. Including a quadratic term in t_B_, in addition to t_B_ itself, did not add significantly to either of value models (professional, youth), nor the frequency models.

Like Vaeyens et al. [37], we found soccer talent was equally distributed throughout the competition year. December born players were equally skilled as January born players, but due to an accident of birth they did not get the breaks, consequently there are fewer of them at the highest levels of football. We therefore agree with Vaeyens et al., (p. 293 in [37]) that, “children disadvantaged by birth date or physical maturity might have become equally skilled senior athletes if they were afforded equivalent developmental opportunities.” But sadly they weren't.

## Supporting information

S1 Table Data for Studies 1 and 2. (XLSX)
